# Serum and urinary soluble CD163 in children with active lupus nephritis

**DOI:** 10.1186/s12969-026-01201-y

**Published:** 2026-03-24

**Authors:** Rasha Hassan El Owaidy, Magid Ashraf Ibraheem, Yasmeen Hasan Alkhateeb, Alaa Adel Taha, Sara I. Taha, Dina E. Sallam

**Affiliations:** 1 Pediatric Allergy, Immunology, and Rheumatology Unit, Faculty of Medicine, Ain Shams University, Cairo, Egypt; 2 Faculty of Medicine, Ain Shams University, Cairo, Egypt; 3 Faculty of Medicine, Al-Azhar University for Girls, Cairo, Egypt; 4 Faculty of Medicine, Alexandira University, Cairo, Egypt; 5 Department of Clinical Pathology, Faculty of Medicine, Ain Shams University, Cairo, Egypt; 6 Pediatrics and Pediatric Nephrology Department, Faculty of Medicine, Ain Shams University, Cairo, Egypt

**Keywords:** Active lupus, BILAG-2004, Lupus nephritis, SLE, SLEDAI, Soluble CD163

## Abstract

**Background:**

Lupus nephritis (LN) is a major contributor to morbidity and death in systemic lupus erythematosus (SLE), particularly in children. CD163 is a macrophage- and monocyte-specific receptor that can be cleaved and released as soluble CD163 (sCD163). Both circulating and urinary sCD163 reflect macrophage activation and are elevated in inflammatory and autoimmune conditions, highlighting their potential as biomarkers of renal inflammation. There is unmet need to develop a set of biomarkers that can accurately reflect disease activity and prognosis and differentiate active from non-active cases. **In this context**, we aimed to evaluate serum and urinary sCD163 levels in children with active LN and to examine their association with established clinical and laboratory markers of SLE activity. Additionally, we explored differences in sCD163 levels between patients with renal versus extrarenal SLE activity and between inflammatory LN and non-inflammatory chronic kidney disease (CKD).

**Methods:**

We enrolled 60 pediatric participants in four qual groups, Group 1: patients with active LN, group 2: SLE non-LN with active extrarenal disease, determined by SLEDAI and BILAG-2004 indices, group 3: non-lupus CKD, and group 4: healthy matched controls. Serum and urine levels of sCD163 were determined in all participants by ELISA.

**Results:**

Serum and urinary sCD163 levels differed significantly among the study groups, with the highest levels in active LN. Patients with active renal BILAG scores A or B had significantly higher sCD163 levels than those with BILAG E, while no significant differences across histopathological classes. Serum sCD163 correlated negatively with eGFR and positively with proteinuria, whereas urinary sCD163 correlated positively with SLEDAI, proteinuria, and prednisolone dose. Both serum and urinary sCD163 distinguished renal from extrarenal SLE activity and LN from non-inflammatory CKD, with complete separation between groups (AUC = 1). No significant correlation was found between serum and urinary sCD163.

**Conclusion:**

Both sCD163 levels were associated with LN activity and differed from levels in extrarenal SLE and non-inflammatory CKD, suggesting that sCD163 may serve as a potential biomarker of kidney inflammation in pediatric SLLE patients.

## Introduction

Lupus nephritis (LN) is a serious and common manifestation of systemic lupus erythematosus (SLE) and a major risk factor for morbidity and mortality [1]. About 60% of SLE patients develop LN, and 10–20% of them progress to end stage kidney failure [2]. Active LN and CKD share a common presentation of proteinuria and impaired kidney function. Differentiation between them is essential for determining the proper management plan and avoiding unnecessary immunosuppression [3]. While kidney biopsy remains the gold standard for diagnosing LN and assessing disease activity and chronicity, its invasive nature, cost, and limited feasibility for repeated assessments restrict its routine use for longitudinal follow-up of renal disease activity [4]. There is no universally agreed-upon biomarker that can accurately and solely reflect lupus kidney activity or damage status, with a current unmet need for a sensitive and specific biomarker. Active kidney inflammation in LN is conventionally assessed using clinical and laboratory markers such as proteinuria, abnormal urine sediment, and changes in serum creatinine or estimated glomerular filtration rate (eGFR). Importantly, proteinuria often precedes a decline in eGFR, and active LN may occur despite preserved kidney function [5]. However, urinary protein quantification cannot differentiate between SLE disease activity and CKD whether related to SLE or other disorders (e.g., diabetes, amyloidosis, drug induced kidney disorders) [6]. In addition, current serum biomarkers, including serum complements (C3 and C4) and anti-double-stranded DNA (anti-ds DNA) antibodies, although are commonly affected in active LN, yet they are not specific to kidney activity and again cannot differentiate kidney activity from damage nor able to predict the histopathological class of LN [7].

CD163 is a 130-kDa endocytic transmembrane protein, a member of the cysteine-rich scavenger receptor superfamily type B originally described as a scavenger receptor for hemoglobin haptoglobin complexes and is expressed solely on macrophages and monocytes [8]. As a result of ectodomain shedding, the extracellular portion of CD163 circulates in blood as a soluble protein (sCD163) at a serum level of 0.7–3.9 mg/L in healthy individuals. During inflammation and macrophage activation, sCD163 levels increased acutely due to metalloproteinase-mediated cleavage near the cell membrane. Thus, it can be considered as a useful biomarker reflecting macrophage activation in various inflammatory diseases, such as macrophage activation syndrome (MAS), autoimmune disorders, sepsis, and liver disease. Moreover, sCD163 could be shed into the urine in active kidney diseases [9, 10]. On this basis, the current study aimed to evaluate serum and urinary sCD163 levels in children with active LN and to examine their association with established clinical and laboratory markers of SLE activity. Additionally, we aimed to explore differences in sCD163 levels between patients with renal and extrarenal SLE activity and between inflammatory LN and non-inflammatory chronic kidney disease.

## Methods

### Ethical considerations

The current study was carried out after approval by the Research Ethics Committee (REC) of the Ain Shams University Faculty of Medicine (***approval numbers: FMASU MS 740/2021 and 758/2021***). A written informed consent in simple Arabic language was obtained from all the participants and from the parents/legal guardians of participants aged 16 years and below, prior to enrollment in the study. All data were kept private and utilized exclusively for research in accordance with the Declaration of Helsinki.

### Study participants

This pilot, controlled, cross-sectional study included 60 pediatric participants with active LN and active extrarenal SLE, and controls groups (< 18 years), who were recruited from the Pediatric Immunology, Rheumatology, Nephrology Units and Outpatient Clinic, at the Children’s Hospital, Faculty of Medicine, Ain Shams University, Cairo, Egypt.

### Clinical evaluation

All participants underwent a comprehensive medical history review and detailed general and systemic examination, with anthropometric measurements and blood pressure recorded. All SLE patients were further assessed for lupus-related manifestations, including constitutional symptoms, carditis, serositis, synovitis, neurological, musculoskeletal, hematological, cutaneous, and kidney involvement, as well as disease duration and activity, disease, and prior management.

### Disease activity for SLE

All included SLE patients were first evaluated at enrollment for disease activity using the SLE Disease Activity Index (SLEDAI) [11] that evaluated the overall disease activity based on clinical and laboratory assessments within 10 days prior to enrollment, and for kidney involvement using Renal BILAG-2004 Index, within 30 days prior to enrollment, which is a validated tool assessing kidney disease activity in SLE based on clinical and laboratory evaluations and the principle of intention to treat. The parameters including proteinuria, blood pressure, urinary sediment, kidney function, and presence of nephrotic syndrome, which is converted into numerical scores (e.g., A = 12, B = 8, C = 1, D/E = 0) to enable longitudinal assessment of disease activity and treatment response, where grade A is highly active kidney disease requiring intensive immunosuppression, grade B is moderate activity, grade C is mild activity, grade D is inactive but previously active disease, and grade E no renal involvement [11, 12].

### Laboratory investigations

Results of recent (within 10 days) routine laboratory investigations performed by the standard methods at Ain Shams University, Clinical Pathology Department, had been collected from patients’ medical records, including: complete blood count (CBC) with differential using Sysmex XT-1800i autoanalyzer (Sysmex, Japan), serum creatinine, blood urea, and 24-hour urinary protein quantification using AU680 Beckman Coulter autoanalyzer (Beckman Coulter, Inc., Brea, CA), erythrocyte sedimentation rate (ESR) using Westergren method [15], anti-nuclear (ANA) and anti-dsDNA antibody titer using indirect immunofluorescence technique (slides by Inova Diagnostics, Inc., San Diego, USA), complement C3 and quantitative C-reactive protein (CRP) levels using COBAS C311 autoanalyzer (Roche Diagnostics, Switzerland). In addition, eGFR according to Schwartz formula was measured and corrected according to the body surface area [16].

From each participant, a random urine sample was collected into a sterile container and a three milliliters blood sample collected by a sterile venipuncture into a plain vacutainer tube were obtained once at the same time. Blood samples were allowed to clot then the clotted blood and urine samples were centrifuged at 2500 rpm for 20 min. Separated sera and urine supernatants were stored at -80°c until further analysis of sCD163 by a human ELISA kit (Bioassay Technology Laboratory, China, Cat no.: E0246Hu). Results of sCD163 were expressed in ng/mL.

### Kidney biopsy

Medical records were revised for the results of kidney biopsies of the recruited LN participants whenever done within 3 to 6 months before enrolment. The biopsies were examined by light, immunofluorescence and electron microscopy and results were interpreted according to the 2003 International Society of Nephrology (ISN)/Kidney Pathology Society (RPS) Classification of LN [13] with the determination of activity and chronicity indices by the scoring system for LN activity and chronicity [14]. Kidney biopsies of participants in group 3 were examined properly using suitable techniques and stains based on the pathological findings.

All participants were equally allocated into four groups (15 participants per group):

### Groups 1 and 2: SLE patients

- **Group 1**: SLE patients with active LN, defined by renal BILAG-2004 score of A or B (within 30 days before enrolment) [12].
- **Group 2**: SLE patients with active extrarenal disease, defined by a recent increase in SLEDAI score of ≥ 3 (within 10 days) [11], and with no kidney activity based on renal BILAG-2004 score D or E (within 30 days) [12].

### Groups 3 and 4 served as control groups

- **Group 3**: patients with CKD not on hemodialysis (HD) with histologically proven chronic tubulointerstitial or ciliopathy-related kidney disease (such as nephronophthisis and Joubert syndrome –associated nephronophthisis), within 3 to 6 months before enrollment, and explicitly excluded primary glomerular diseases (e.g., IgA nephropathy) to reduce pathological heterogeneity within the group.
- **Group 4**: age and gender matched healthy controls.

Patients were excluded if they had clinical or laboratory features suggestive of macrophage activation syndrome (MAS), autoimmune diseases other than SLE (e.g., mixed connective tissue disease, juvenile idiopathic arthritis, dermatomyositis, systemic vasculitis), or active infection within 30 days of the time of enrollment. Patients with CKD on HD were also excluded. Additionally, individuals who had not undergone a kidney biopsy within 3 to 6 months were excluded from groups 1 and 3.

### Statistical methods

Data were analyzed using IBM© SPSS© Statistics version 26 (IBM© Corp., Armonk, NY) and MedCalc^®^ Statistical Software version 20 (MedCalc Software Ltd, Ostend, Belgium; https://www.medcalc.org; 2021). Normally distributed numerical variables were presented as mean and SD and inter-group differences were compared using one-way analysis of variance (ANOVA) with application of Dunnett’s test for post-hoc comparisons when needed. Skewed numerical data were presented as median and interquartile range and differences were compared using the Kruskal Wallis test with application of the Conover post hoc test when needed. For comparisons between two independent groups, the Mann–Whitney U test was applied. Categorical variables were presented as counts and percentages and compared using the Pearson chi-squared test or Fisher’s exact test, as appropriate. Ordinal data were compared using linear-by-linear association. Correlations between numerical or ordinal variables were tested using the Spearman rank correlation. Correlations between numerical or ordinal variables were tested using the Spearman rank correlation. Point-biserial correlation is used to examine association between numerical and binary variables. The correlation coefficients (Spearman’s rho or point biserial correlation coefficient) were interpreted as follows: <0.2 = very weak, 0.2 to 0.39 = weak, 0.4 to 0.59 = moderate, 0.6 to 0.79 = strong, ≥ 0.8 = very strong.

Response-operating characteristic (ROC) curve analysis was used to examine the discriminative value of sCD163. The area under the ROC curve (AUC) was interpreted as follows: AUC < 0.6 = fail, 0.6 to 0.69 = poor, 0.7 to 0.79 = fair, 0.8 to 0.89 = good, ≥ 0.9 = excellent. For the Conover test, the significance level was set at *p* < 0.008 to adjust for multiple pair-wise comparisons. Otherwise, p-values < 0.05 were considered statistically significant. To construct confidence intervals for the ROC analysis, we employed the bias-corrected and accelerated (BCa) bootstrap method. The BCa intervals were calculated using 1,000 bootstrap replications, as recommended for stable estimates. This methodology follows the framework described by ***Efron and Tibshirani*** [35] and was implemented using the MedCalc statistical software, version 22.009 (MedCalc Software Ltd, Ostend, Belgium).

## Results

The clinico-demographic and laboratory characteristics of the studied groups are shown in Tables 1 and 2. Female predominance was noted among lupus patients while male gender prevailed group 3 patients. Compared to controls, patients in groups (1), (2), and (3) tended to have shorter stature (*p* < 0.001). Group (3) showed a statistically significant lower BMI values compared to other groups (*p* < 0.001). Patients in groups (1), (2), and (3) all had hypertension, at rates of 33.3%, 40%, and 100%, respectively. The most frequent histological class found in kidney biopsies among patients with active LN (group 1) was class V (7/15), followed by class III (4/15), class IV (2/15), and class II (2/15). Patients in group 3 were identified by kidney biopsy as chronic tubulointerstitial nephritis (8/15), nephronophthisis (5/15), and Joubert syndrome (2/15).

**Table 1:** Comparison of demographic, clinical, and laboratory characteristics of all participants

Variable	Group (1) (no.=15)	Group (2) (no.=15)	Group (3) (no.=15)	Group (4) (no.=15)	*p*-value
Age (years) Mean (± SD)	12.8 ± 2.9	12.8 ± 3.3	10.4 ± 4.2	11.7 ± 2.3	0.146
Gender					
Male	1 (6.7%)	1 (6.7%)	13 (86.7%)	6 (40.0%)	< 0.001
Female	14 (93.3%)	14 (93.3%)	2 (13.3%)	9 (60.0%)	
Weight percentile					
< 5th	0 (0.0%)	0 (0.0%)	4 (26.7%)	0 (0.0%)	0.072
Normal	10 (66.6%)	13 (86.7%)	11 (73.3%)	14 (93.4%)	
> 95th	5 (33.3%)	2 (13.4%)	0 (0.0%)	1 (6.7%)	
Height percentile					
< 5th	12 (80.0%)	13 (86.7%)	8 (53.3%)	0 (0.0%)	< 0.001
Normal	3 (20.0%)	2 (13.3%)	7 (46.7%)	14 (93.3.0%)	
> 95th	0 (0.0%)	0 (0.0%)	0 (0.0%)	1 (6.7%)	
BMI (kg/m^2^)	28.0 ± 6.6	26.4 ± 6.4	15.9 ± 2.5	22.6 ± 3.0	< 0.001
Hypertension	5 (33.3%)	6 (40.0%)	15 (100.0%)	0 (0.0%)	< 0.001
Laboratory Data					
TLC (k/mm^3^)	7.3 ± 3.1	6.7 ± 1.7	12.7 ± 3.6	5.5 ± 1.2	< 0.001
ANC (k/mm^3^)	3.1 ± 1.8	3.3 ± 1.2	8.4 ± 2.4	3.7 ± 1.1	< 0.001
ALC (k/mm^3^)	1.9 ± 0 0.7	2.9 ± 1.3	3.3 ± 1.3	1.4 ± 0.5	< 0.001
Hemoglobin (g/dl)	11.7 ± 1.8	10.0 ± 1.3	9.0 ± 1.3	12.2 ± 1.1	< 0.001
Platelets (k/mm^3^)	301 ± 77	219 ± 86	147 ± 95	254 ± 32	< 0.001
ESR (mm)	60 (23–93)	35 (16–87)	43 (40–45)	9 (6–13)	< 0.001
Positive CRP	8 (53.3%)	6 (40.0%)	15 (100.0%)	0 (0.0%)	< 0.001
Serum creatinine (mg/dl)	0.51 ± 0.14	0.47 ± 0.07	2.24 ± 0.93	0.49 ± 0.11	< 0.001
eGFR (ml/min/1.73 m^2^)	116.5 ± 38.0	120.1 ± 19.0	28.6 ± 14.6	129.2 ± 20.1	< 0.001
Blood urea (mg/dl)	18.0 ± 4.5	20.1 ± 4.2	63.5 ± 17.7	11.0 ± 4.1	< 0.001
Urinary protein (g/24 h)	0.45 (0.33–0.85)	0.17 (0.14–0.22)	0.63 (0.37–0.77)	0.19 (0.14–0.21)	< 0.001

Abbreviations: ALC, absolute lymphocyte count; ANC, absolute neutrophil count; BMI, body mass index; CRP, C-reactive protein; ESR, erythrocyte sedimentation rate; eGFR, estimated glomerular filtration rate; LN, lupus nephritis; SLE, systemic lupus erythematosus; TLC, total leucocytic count. Group (1): SLE with active LN, group (2): SLE with non-kidney activity, group (3): non-lupus kidney disease, group (4): Healthy control

**Table 2:** The main disease clinico-demographic parameters and laboratory data among 30 recruited patients with SLE (**groups 1 and 2**) (*n* = 30)

Variables	Value
Age at diagnosis of SLE (years)	10.7 ± 3.1 (5.0–15.6)
Duration of SLE (years)	2.1 ± 1.7 (0.1–7.0)
Duration of LN (years)	2.1 ± 1.4 (0.2–5.0)
SLE SLEDAI	12 ± 8 (4–34)
**Kidney BILAG − 2004 Index**	
BILAG A	4/30 (13.3%)
BILAG B	11/30 (36.7%)
BILAG E	15/30 (50.0%)
**Extra kidney manifestations**	
Arthritis	28/30 (93.3%)
Constitutional manifestations	25/30 (83.3%)
Hematological manifestations	19/30 (63.3%)
Carditis	16/30 (53.3%)
**Specific immunological investigations**	
Positive ANA	30/30 (100.0%)
Positive Anti-dsDNA	28/30 (93.3%)
C3 (mg/dl)	98 ± 44 (27–177)
Consumed C3	13 (43.3%)
Medications	
**Prednisolone dose (mg/kg/day)**	0.3 ± 0.1 (0.2–0.5)
Cyclophosphamide	5 (16.7%)
Tacrolimus	1 (3.3%)
HCQ	22 (73.3%)
MMF	15 (50.0%)

Data are presented as the number of patients (%) and mean ± SD (minimum-maximum)

Abbreviations: ANA, antinuclear antibodies; Anti-dsDNA, anti- double stranded DNA; BILAG, British Isles Lupus Assessment Group; CKD, chronic kidney disease; HCQ, hydroxychloroquine; LN, lupus nephritis; MMF, mycophenolic acid; SLE, systemic lupus erythematosus; SLEDAI, systemic lupus erythematosus disease activity index

**Fig. 1:**
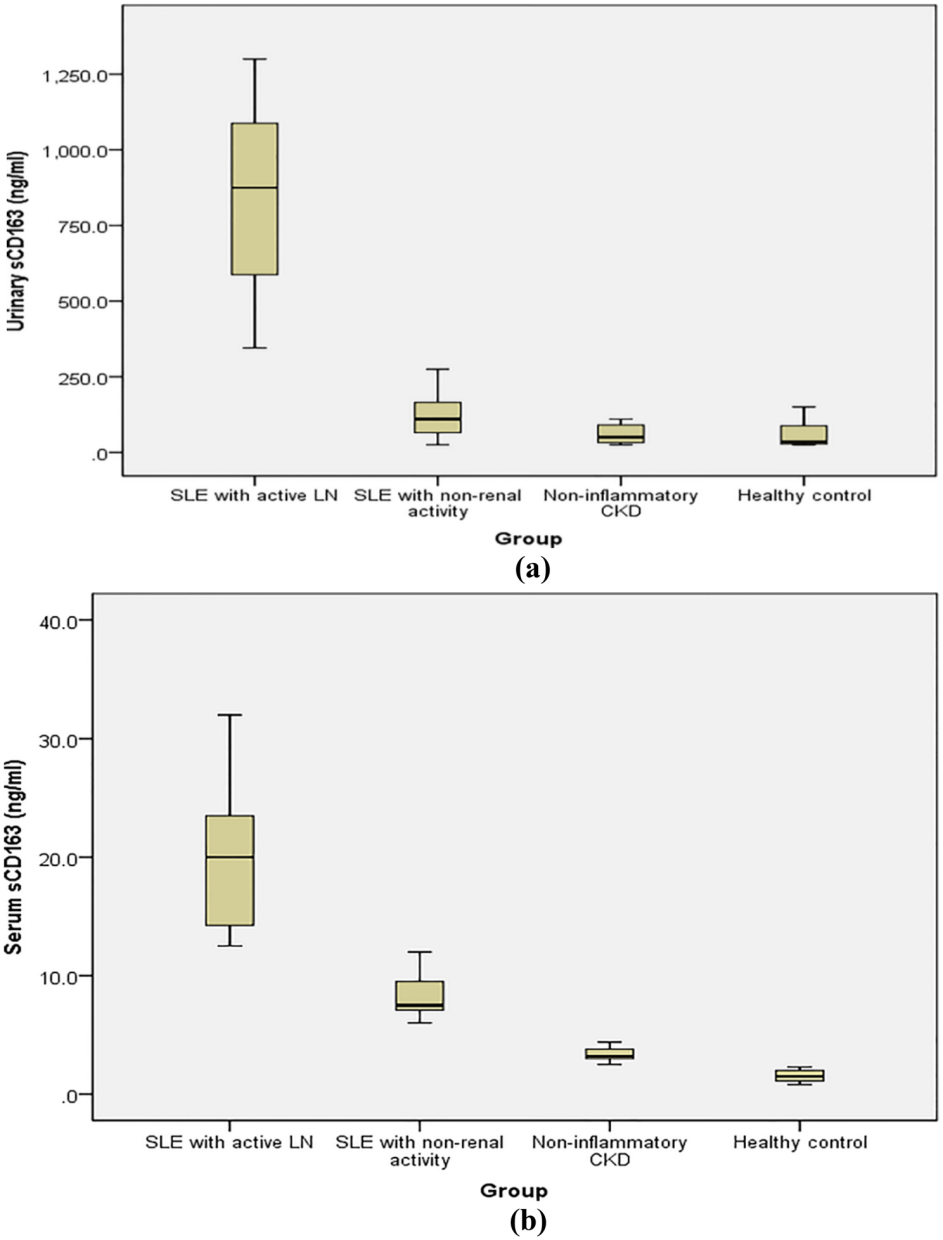
Box plots illustrating urinary (**a**) and serum (**b**) sCD163 levels in the four studied groups. Box represents interquartile range. The line inside the box represents the median. Error bars represent minimum and maximum values

Levels of serum and urine sCD163 differed significantly (*p*-values < 0.001) between the studied groups, being the highest in group 1. The median (IQR) serum sCD163 was 20 (14.1–24.3) ng/mL in group 1, 7.5 (7.1–9.5) ng/mL in group 2, 3.2 (3.0-3.8) ng/mL in group 3, and 1.5 (1.1-2.0) in group 4. On the other hand, the median (IQR) urine sCD163 was 875 (568.8–1143.8) ng/mL in group 1, 110 (60–165) ng/mL in group 2, 50 (31.3–90) ng/mL in group 3, and 35 (26.3–93.8) ng/mL in group **4** (Fig. 1**).** Meanwhile, serum and urine sCD163 levels were markedly higher in SLE patients compared with Non-SLE CKD patients. The median serum sCD163 in SLE patients was 12.3 ng/mL (IQR: 7.5–20.0), compared with 3.2 ng/mL (IQR: 3.0–3.8) in Non-SLE CKD patients, a difference that was highly significant (*P* < 0.0001). Similarly, urine sCD163 levels were substantially elevated in SLE patients, with a median of 310.0 ng/mL (IQR: 110.0–875.0) versus 50.0 ng/mL (IQR: 31.3–90.0) in Non-SLE CKD patients (*P* < 0.0001) (Table 3, and Fig. 4).

**Table 3:** Comparison between serum and urine sCD163 levels in SLE (Groups 1 & 2) and Non-SLE CKD (Group 3)

Variable	SLE (*N* = 30)	Non-SLE CKD (*N* = 15)	*P* value *
**Serum sCD163 (ng/mL)** Median (IQR)	12.3 (7.5–20.0)	3.2 (3.0–3.8)	< 0.0001
**Urine sCD163 (ng/mL)** Median (IQR)	310.0 (110.0–875.0)	50.0 (31.3–90.0)	< 0.0001

Abbreviations: CKD: chronic kidney disease, IQR, interquartile range, SLE, systemic lupus erythematosus; *: Mann–Whitney U test used for comparison

**Fig. 2:**
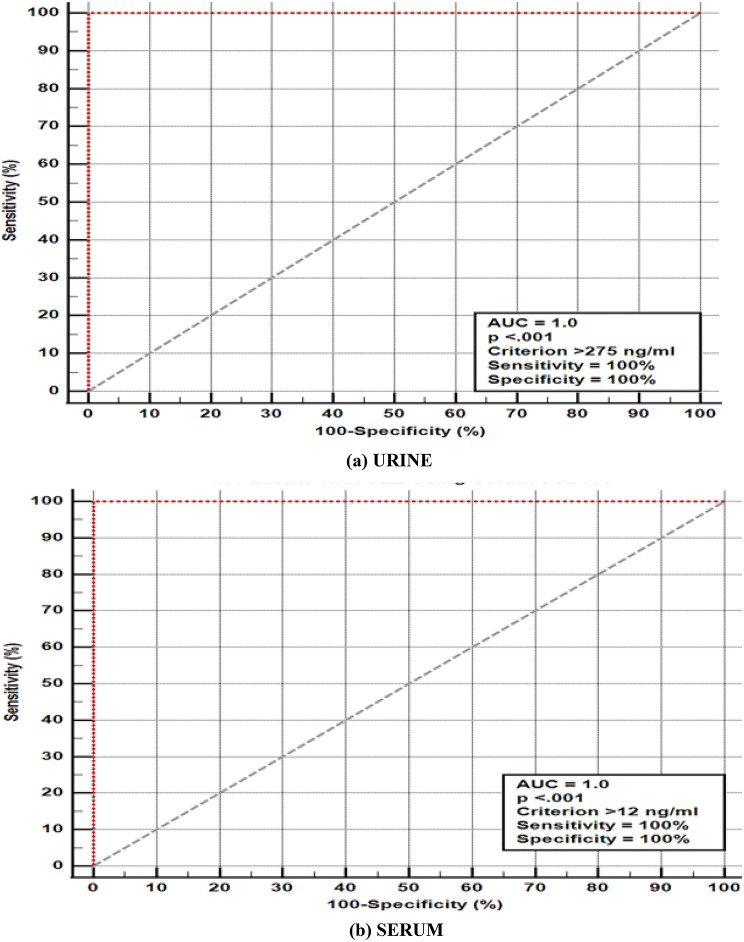
The Receiver-Operating Characteristic (ROC) curve analysis for urinary (**a**) and serum (**b**) sCD163 for discrimination between SLE patients with kidney and non-kidney affection (groups 1 vs. group 2)

**Fig. 3:**
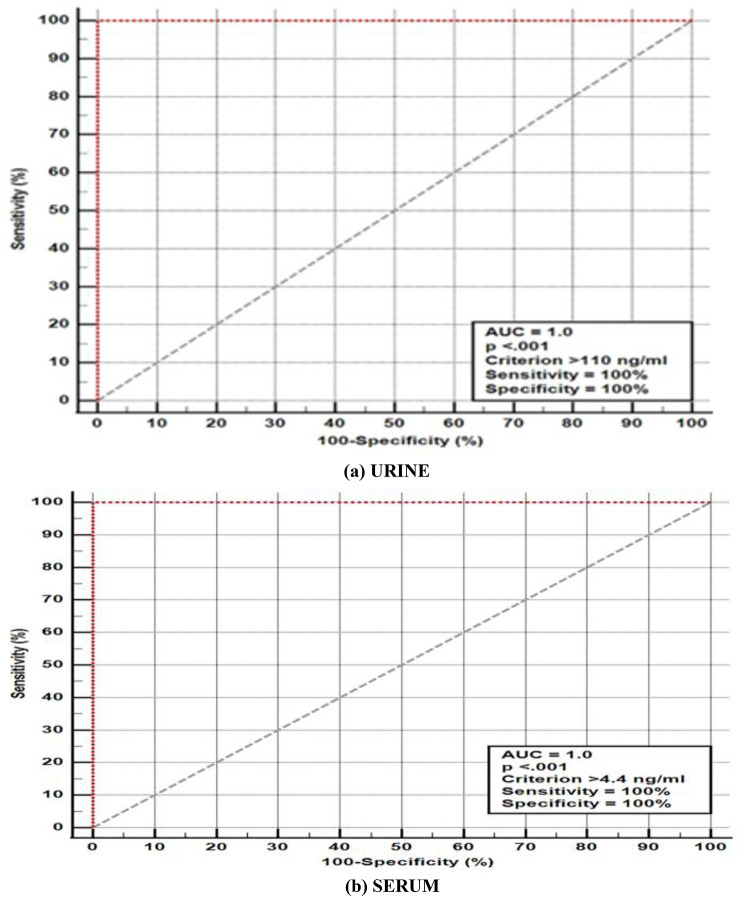
The Receiver-Operating Characteristic (ROC) curve analysis for urinary (**a**) and serum (**b**) sCD163 for discrimination between patients with LN and non-lupus CKD using (group 1 vs. group 3)

**Fig. 4:**
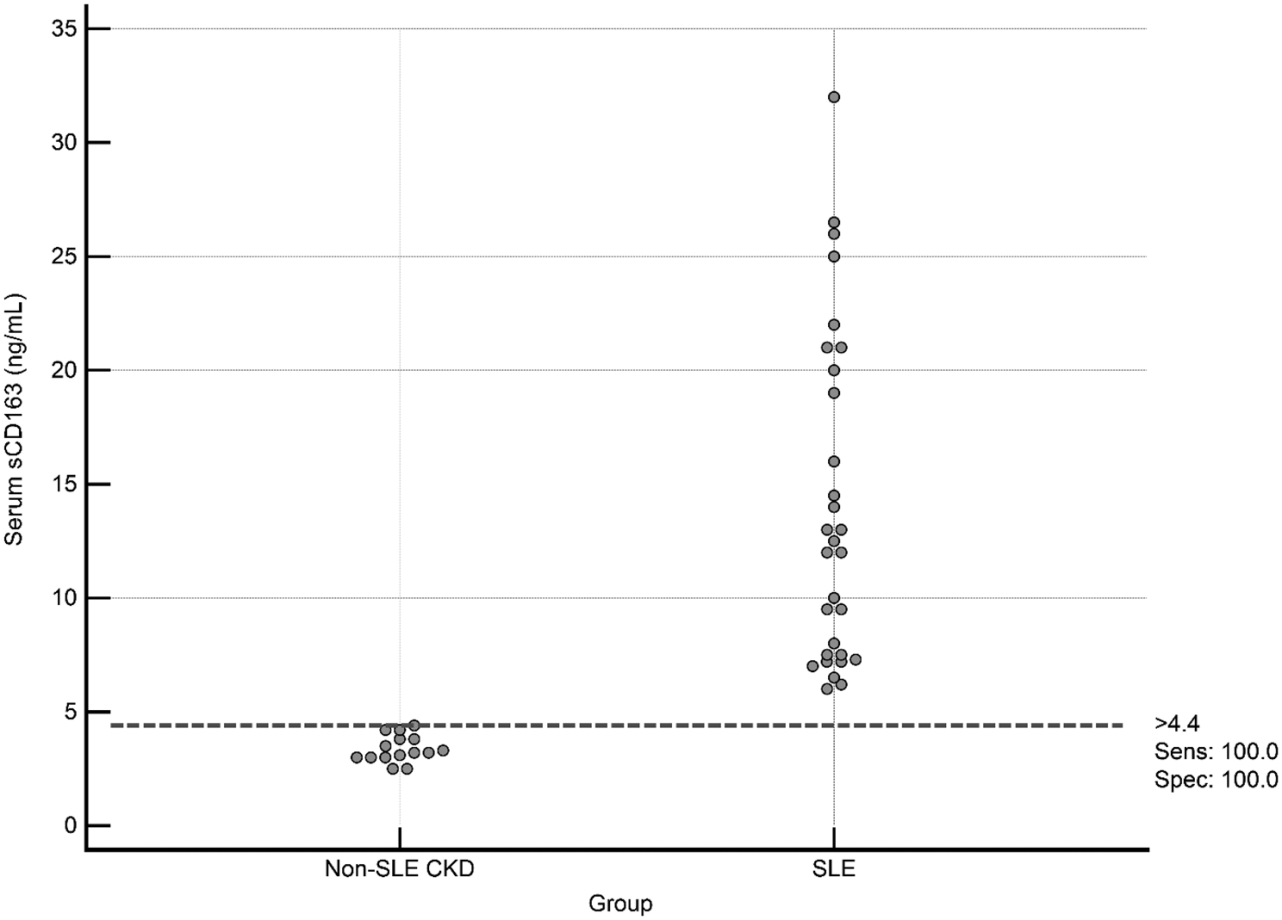
Interactive dot diagram for the discrimination between all SLE patients (no. = 30) and Non-SLE CKD patients (no. = 15) using serum sCD163 level (Groups 1 and 2 Vs Group 3)

**Fig. 5:**
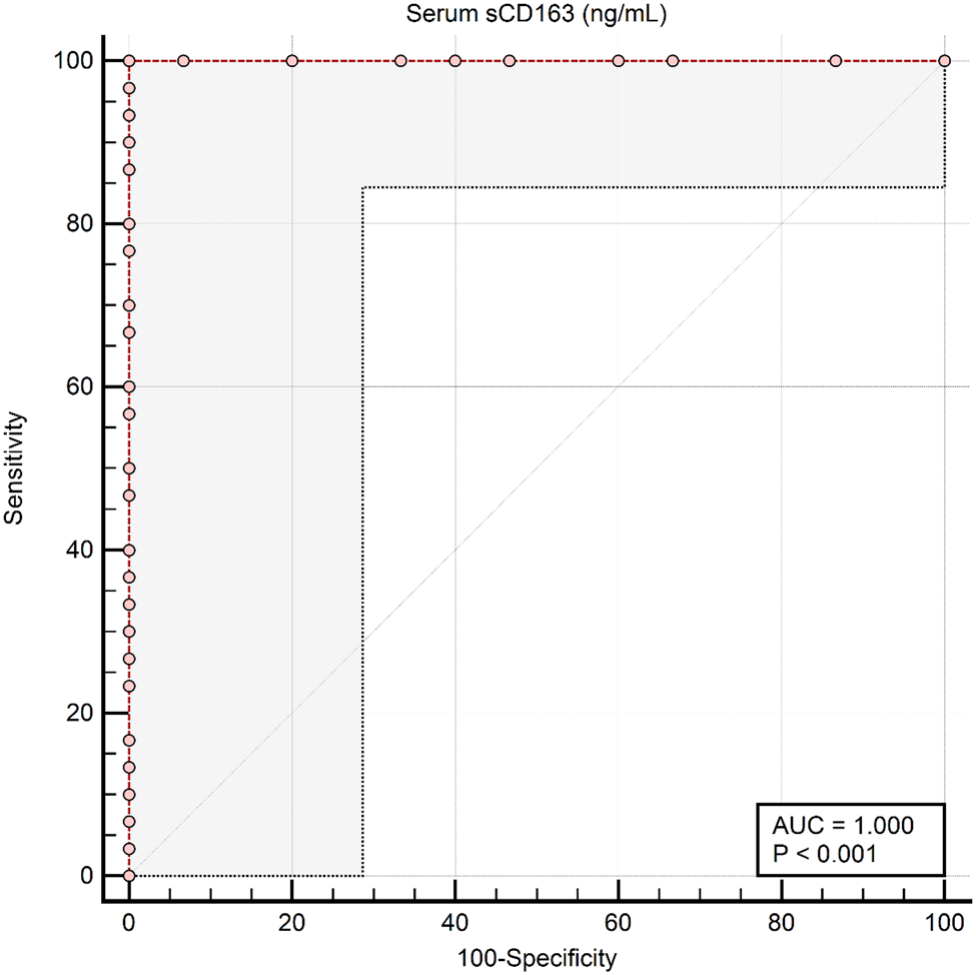
Response-operating characteristic (ROC) curve for the discrimination between all SLE patients (no. = 30) and Non-SLE CKD patients (no. = 15) using serum sCD163 level (Groups 1 and 2 Vs Group 3). Serum sCD163 had perfect discriminative value with an area under the ROC curve (AUC) of 1.0 (bootstrapped 95% CI: 1.0 to 1.0). A cutoff criterion of > 4.4 ng/mL (bootstrapped 95% CI: >4.2 to > 4.4 ng/m) could differentiate between both categories with a sensitivity of 100% and a specificity of 100%

**Fig. 6:**
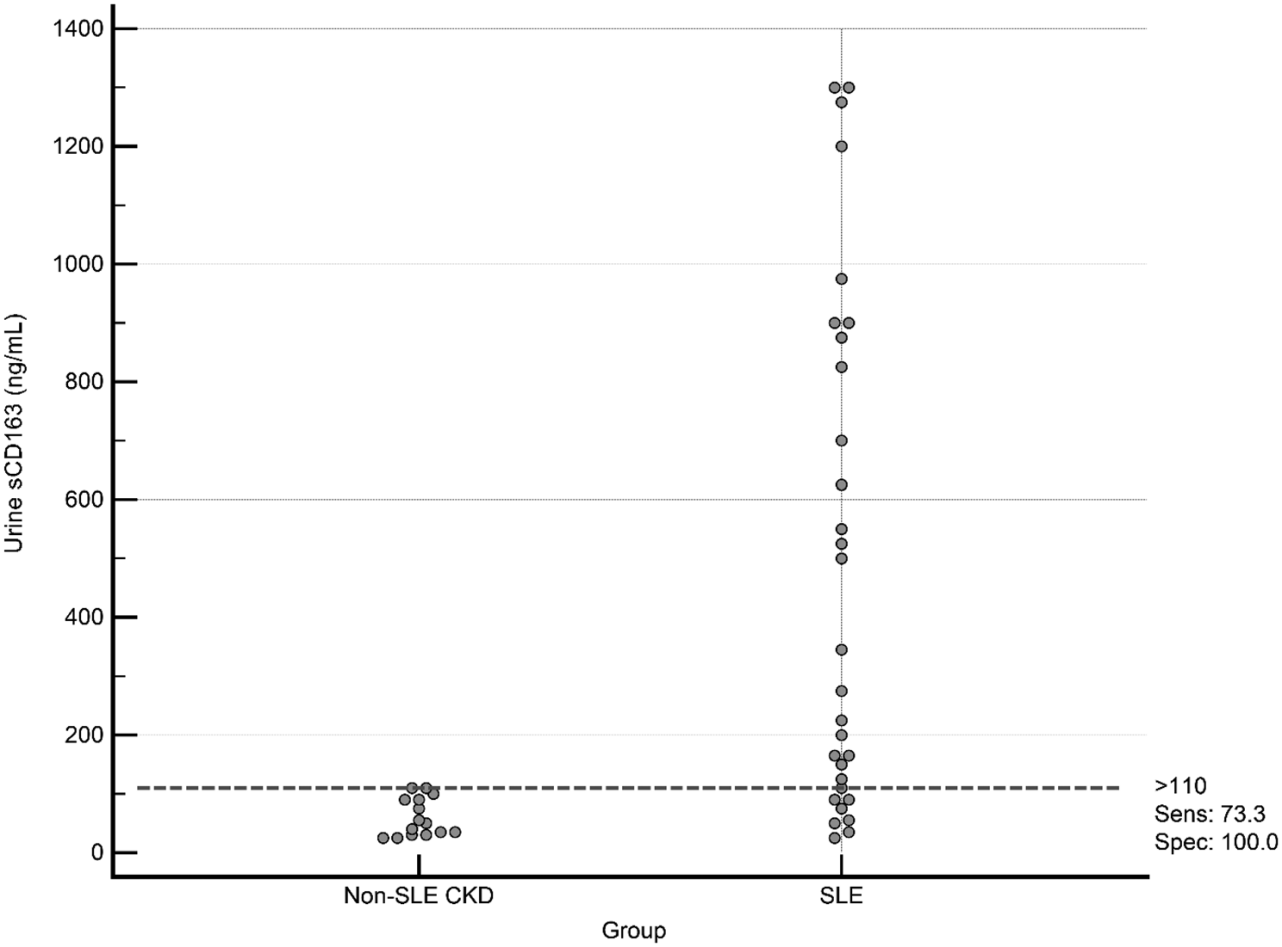
Interactive dot diagram for the discrimination between all SLE patients (no. = 30) and Non-SLE CKD patients (no. = 15) using urine sCD163 level (Groups 1 and 2 Vs Group 3). A cutoff criterion > 110 ng/mL (bootstrapped 95% CI: >90 to > 110 ng/mL) could differentiate between both categories with a sensitivity of 73.3% and a specificity of 100%

**Fig. 7:**
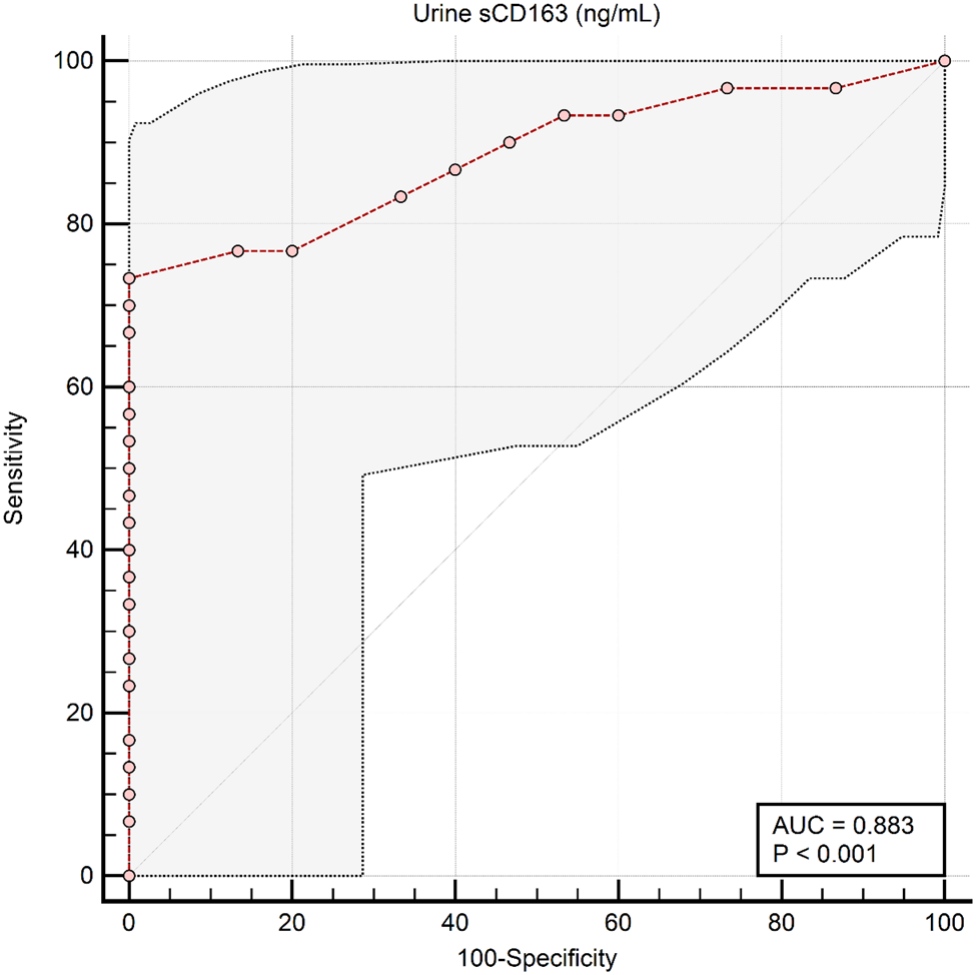
Response-operating characteristic (ROC) curve for the discrimination between all SLE patients (no. = 30) and Non-SLE CKD patients (no. = 15) using urine sCD163 level (Groups 1 and 2 Vs Group 3). Urine sCD163 had good discriminative value with an area under the ROC curve (AUC) of 883, (bootstrapped 95% CI: 0.781 to 0.959). A cutoff criterion > 110 ng/mL (bootstrapped 95% CI: >90 to > 110 ng/mL) could differentiate between both categories with a sensitivity of 73.3% and a specificity of 100%

**Fig. 8:**
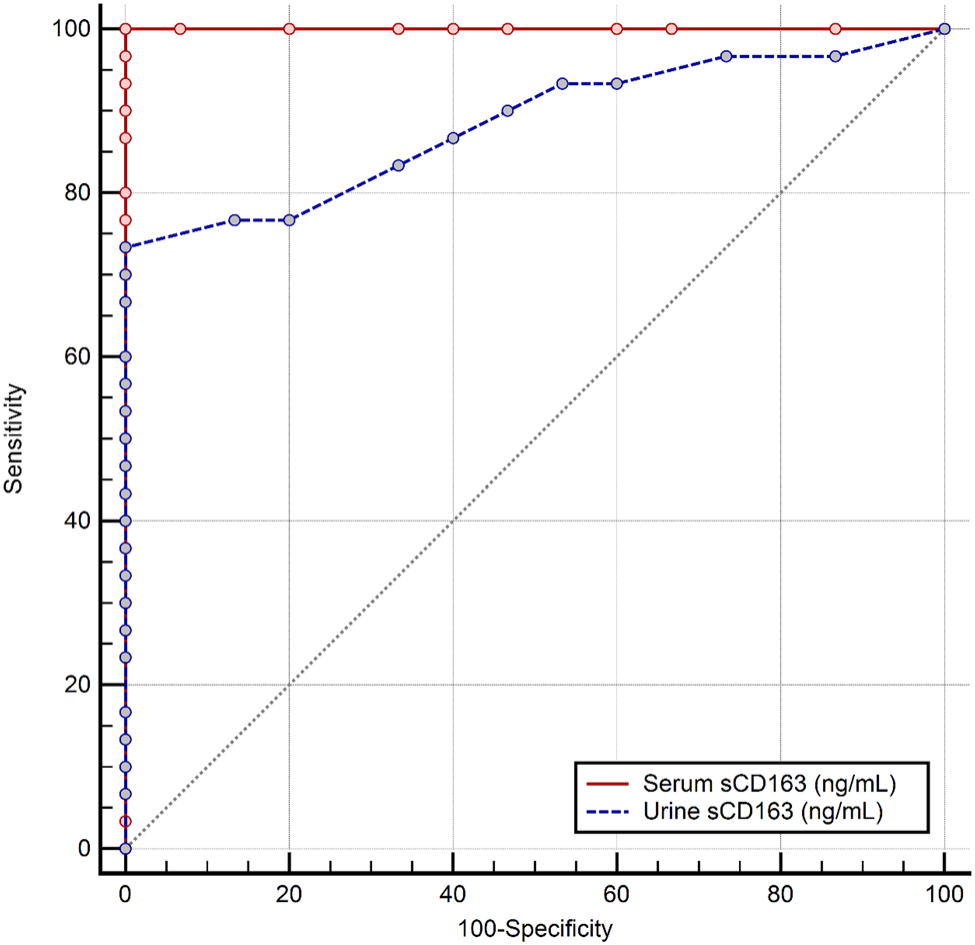
Comparison of the ROC curves associated with serum or urine sCD163 Groups 1 and 2 Vs Group 3). The difference between the area under either ROC curve is statistically significant (ΔAUC: 0.117, 95% CI: 0.021 to 0.213, *P* = 0.017)

Levels of serum and urinary sCD163 did not differ significantly between proliferative (Class III/IV) LN group and non-proliferative (Class II) LN in group 1 (*p*-values = 0.430, and 0.74 respectively) (Table 4). On the other hand, lupus patients with kidney BILAG A and B scores had higher serum and urinary levels of sCD163 compared to those with BILAG E (*p* < 0.001) (Table 5). Among the SLE patients collectively (groups 1 and 2), serum sCD163 showed a significant negative correlation with the eGFR (*r*=-0.382, *p* = 0.037) and a significant positive correlation (*r* = 0.693, *p* < 0.001) with the 24-hour urinary protein but did not correlate with SLEDAI scores (*r* = 0.312, *p* = 0.093). On the other hand, urinary sCD163 levels showed significant positive correlations with SLEDAI score (*r* = 0.394, *p* = 0.031), prednisolone dose in mg/kg/day (*r* = 0.395, *p* = 0.031), and the 24-hour urinary protein (*r* = 0.552, *p* = 0.002).

**Table 4:** Comparison of urinary and serum sCD163 in non-proliferative (Class II) and proliferative (Class III/IV) LN group

Variable	Proliferative nephritis (class III or IV) (no.=6)	Class V nephritis (no.=7)	*p*-value*
Urinary sCD163 (ng/mL) median (IQR)	1013 (500–1300)	875 (550–900)	0.430
Serum sCD163 (ng/mL) median (IQR)	20.5 (19.0–26.0)	16.0 (13.0–22.0)	0.741

Abbreviations: IQR, interquartile range, *: Mann–Whitney U test used for comparison

**Table 5:** Comparison of urinary and serum sCD163 in SLE patients (groups 1 and 2) according to the kidney BILAG-2004 index

	Kidney BILAG			*p*-value
	A (*n* = 4)	B (*n* = 11)	E (*n* = 15)	
Urinary sCD163 (ng/mL) Median (IQR)	950.0 (662.5 − 1250.0)	875.0 (537.5–937.5)	110.0 (65.0–165.0)	< 0.001
Significance between groups	p1 = 0.715, p2 = 0.001, p3 < 0.001			
Serum sCD163 (ng/mL) Median (IQR)	17.25 (13.5–22.5)	21.0 (15.0–24.0)	7.50 (7.1–9.5)	< 0.001
Significance between groups	p1 = 0.690, p2 = 0.006, p3 < 0.001			

Abbreviations: BILAG, British Isles lupus assessment group; IQR, interquartile range

p: p value for comparison between the studied categories

p_1_: p value for comparison **A** and **B**

p_2_: p value for comparison **A** and **E**

p_3_: p value for comparison **B** and **E**

Serum and urinary sCD163 levels differed significantly between patients with renal and extrarenal SLE activity (cut off serum and urine levels > 12 ng/mL and > 275 ng/mL, respectively), and between patients with LN and those with non-inflammatory CKD in this study cohort (cut off > 4.4 ng/mL and > 110 ng/mL, respectively), with AUC = 1 and *p* < 0.001, sensitivity and specificity 100% in all cases. Cut-off values derived from ROC analysis demonstrated complete separation between the studied groups; however, these findings reflect group-level differences within a limited and pre-defined sample rather than diagnostic accuracy (Figs. 2 and 3).

It is worth noting that there was no significant correlation between serum and urinary levels of sCD163 across the studied groups, with p-values of 0.36, 0.88, 0.48, and 0.42 for groups 1, 2, 3, and 4, respectively.

We compared **all SLE patients** (Groups 1 and 2, *n* = 30) with **Non-SLE CKD patients** (Group 3, *n* = 15) to evaluate serum and urine sCD163 as discriminative markers. **Serum sCD163** demonstrated excellent discriminative performance, achieving a perfect area under the ROC curve (AUC = 1.0, 95% CI: 1.0–1.0). A cutoff value of > 4.4 ng/mL perfectly separated SLE from non-SLE CKD patients, with **100% sensitivity and 100% specificity** (Figs. 4 and 5). **Urine sCD163**, in contrast, showed good but slightly lower discriminative ability (AUC = 0.883, 95% CI: 0.781–0.959). A cutoff of > 110 ng/mL correctly identified 73.3% of SLE patients while maintaining 100% specificity (Figs. 6 and 7). Comparison of the ROC curves revealed that serum sCD163 outperformed urine sCD163 in distinguishing SLE from non-SLE CKD, with a statistically significant difference in AUC (ΔAUC = 0.117, 95% CI: 0.021–0.213; *P* = 0.017) (Fig. 8).

## Discussion

Early recognition and accurate assessment of kidney involvement in SLE patients significantly impact long-term prognosis. Although kidney biopsy is the current gold standard for the diagnosis and classification of LN, it is invasive and cannot be done repeatedly to assess response to treatment. Therefore, there is a need for non-invasive biomarkers that may reflect inflammatory renal activity and provide biological insight into disease mechanisms.

The current study revealed that urine and serum sCD163 levels were higher in patients with SLE, either with or without LN, compared to other groups, suggesting an association with systemic and renal inflammatory activity rather than disease specificity. Besides being expressed solely by macrophages and monocytes, the increase in sCD163 can be explained by what Bhattacharya and Aggarwal have reported that the M2 macrophages, which play a key pathogenic role in the development of autoimmune diseases such as SLE, express CD163 and have anti-inflammatory properties; they can help resolve inflammation, remodel tissue, and promote fibrosis [17]. Kopetschke et al., in 2015, reported that urinary macrophages correlate with disease activity and were significantly higher in the active LN group [18]. Moreover, Olmes et al. in 2016, found that activated CD163^+^M2c-like macrophages are abundant in kidney biopsies and urine of LN patients [19]. Several other studies have also shown that the macrophages that infiltrate into LN kidneys are predominantly CD163-expressing cells [20, 21]. Our findings are also in harmony with the study of Yang and their colleagues, who investigated serum sCD163 levels in 121 Eastern Chinese patients with active LN, aged 21–54 years compared to healthy controls. They found that serum sCD163 was significantly higher in patients with active LN [22]. In addition, Zhang et al. 2020, studied the association of urinary sCD163 with proliferative LN in a total of 228 SLE patients and 56 healthy controls from three cohorts from the United States and China and reported that urine sCD163 was significantly higher in active LN patients compared to inactive SLE, active non-kidney SLE, and controls [23]. Similar results were also reported by Gupta et al., [24] and Gamal et al., [25]. On the other hand, Nishino et al. 2019, investigated the serum of 110 adult Japanese SLE patients (32 ± 5 years old) and did not find any significant difference in sCD163 levels between patients of active SLE with or without LN and suggested that the serum levels of sCD163 fail to reflect kidney disease activity [26]. Variations in the age of participants, sample size, and racial factors might have caused this discrepancy.

In our study, though circulating sCD163 levels was influenced by kidney clearance, represented by eGFR, as agreed also with other study by Huang YJ 2022 [27], however the relatively low serum sCD163 observed in group 3, despite the most severe kidney dysfunction, appears contradictory. This suggests that in advanced CKD, factors beyond kidney filtration, such as altered macrophage activation, reduced CD163 shedding due to macrophage exhaustion, or changes in systemic inflammatory status, may play a dominant role [28]. Therefore, while serum sCD163 may reflect disease activity in earlier stages, it may not reliably indicate kidney involvement in advanced CKD. In contrast, urinary sCD163 appears to more accurately reflect ongoing renal inflammation and may represent a more robust biomarker for active LN.

In the current study, the most prevalent histopathological class of LN was class V (46.6%), followed by proliferative nephritis (classes III and IV) (40%), while only 2/15 (13.3%) patients had class II, and patients in these classes were comparable in terms of urine and serum sCD163 levels. Zhang et al. 2020, reported that urine sCD163 was significantly elevated in patients with proliferative LN, especially in class IV LN [23]. We believe that the small sample size and the inadequate representation of different classes of LN might have limited our findings. However, Gupta et al. 2021, and Mejia-Vilet et al. 2020, also found that urinary sCD163 levels did not vary significantly according to the histologic class in LN patients [24, 29].

The kidney BILAG-2004 score reflects changes in the kidney parameters, while the kidney SLEDAI score describes the present abnormalities regardless of the change in them over the period before assessment [11, 12]. In the current study, urine and serum sCD163 were significantly higher in SLE patients with kidney BILAG A and B compared to BILAG E but were comparable between BILAG A and BILAG B patients. Both BILAG A and B scores represent kidney activity, while BILAG E represents quiescent or absent kidney affection [12]. These results agree with *Zizzo et al. 2013*, who measured levels of several blood biomarkers in 107 SLE patients aged between 19 and 51 years and found that the highest values of serum sCD163 were observed in patients with more active LN (BILAG A) [30]. Also, *Zhang et al. 2020*, reported that urine sCD163 strongly correlated with kidney BILAG-2004 and activity index of kidney pathology [23].

Additionally, in the current study, despite the significant positive correlation between urine sCD163 levels and SLEDAI scores in SLE patients (groups 1 and 2), the positive correlation between serum sCD163 and SLEDAI scores did not reach statistical significance, this finding can be explained by the inclusion of many factors other than kidney parameters in the SLEDAI grading criteria. These results agree with Yang et al. 2021, who found no statistical difference in SLEDAI scores between the high and normal serum sCD163 lupus patients [22]. In contrast, Zizzo et al. 2013, found that serum sCD163 correlated positively with SLEDAI scores in adult patients with SLE [30]. Other studies also found that urine sCD163 levels were associated with kidney SLEDAI scores [23, 24]. Gamal et al. 2022, observed that urinary sCD163 had significant correlations with total and kidney SLEDAI scores but not with extrarenal SLEDAI, which suggests that urinary sCD163 level in SLE patients is not a marker of systemic inflammation but rather reflects kidney activity [25].

In a recently published article by Renaudineau et *al.*, a monocentric and retrospective study was conducted on 139 SLE patients with biopsy-proven nephritis including 63 active LN and 76 inactive LN, as well as 98 non-kidney SLE patients. The authors found that urinary sCD163/creatinuria ratio outperformed the other serological markers for predicting active LN namely anti-dsDNA and anti-C1q antibodies [34].

We noticed significant positive correlations between urine and serum sCD163 with 24-hour urinary protein levels in SLE patients (groups 1 and 2). Consistent with our results, other studies found a significant positive correlation between urine sCD163 and 24-hour protein in urine [24, 29, 31]. Gupta et al. 2021, reported that SLE patients with nephrotic syndrome had higher urine CD163 levels (median 2678 ng/mmol; IQR, 982–6019 ng/mmol) than those with non-nephrotic proteinuria, hematuria, and normal kidney function (760 ng/mmol; IQR, 307–1778 ng/mmol, *p* = 0.001) [24].

The current study also found a positive correlation between urine sCD136 levels and prednisolone dose in SLE patients (groups 1 and 2), most probably owing to poor disease control as higher prednisolone doses were given to patients with higher activity status. Other studies supported this finding [27, 32].

In our study, serum and urinary sCD163 levels differed markedly between patients with renal versus extrarenal SLE activity, and between patients with LN and those with non-inflammatory CKD. ROC analysis of this cohort produced cut-off values of serum sCD163 ng/mL and urine sCD163 > 275 ng/mL for differentiating renal from extrarenal SLE activity, and serum sCD163 > 4.4 ng/mL and urine sCD163 > 110 ng/mL for separating LN from non-lupus CKD. Additionally, serum sCD163 showed outstanding discriminative ability to distinguish between patients diagnosed with SLE patients (with LN or without) from those with non-SLE CKD, achieving perfect separation with a cutoff > 4.4 ng/mL, meanwhile, urine sCD163 demonstrated good discriminative performance at a cutoff of > 110 ng/mL. Comparison of ROC curves confirmed that serum sCD163 outperformed urine sCD163, suggesting that serum measurements may be more reliable for clinical differentiation, while urine sCD163 could provide complementary information. These findings support the potential utility of sCD163 as a biomarker for SLE; however, the results reflect group-level separation within pre-defined study groups and should be interpreted as exploratory and associative rather than diagnostic. Further validation in larger, clinically heterogeneous cohorts including inflammatory non-LN is required before any clinical application. Gupta et al. 2021, reported that urine sCD163 could differentiate between active LN and active non-kidney disease better than plasma sCD163 [24]. Meanwhile, the cutoff value of urine sCD163 in the study of Mejia-Vilet et al. 2020, was 130 ng/mmol which had 97% sensitivity and 94% specificity to distinguish between patients with active LN and those with inactive LN [29]. Another study by Dekkema et al. 2019, showed that urine sCD163 at a cut-off value of > 350 ng/mmol differentiated active from inactive LN with > 70% sensitivity and > 94% specificity [33]. Different units of measurement, different kits used, variations in sample size, and the age of participants, their disease characteristics and their kidney histopathological findings are all factors that should be kept into consideration.

The present study has **several limitations**. It employed a cross-sectional design with modest cohort size and consecutive enrollment, and representation across LN classes was unbalanced. An inactive lupus group was not included. Kidney biopsies were not uniformly timed with sCD163 measurement, though were done within 3 to 6 months from study enrollment. The non-LN group consisted of ciliopathy-related and chronic tubulointerstitial kidney disease, which does not represent the full spectrum of inflammatory glomerulonephritis encountered in differential diagnosis, restricting generalizability. Therefore, results should be interpreted as exploratory and associative within the studied populations rather than indicative of diagnostic performance.

**In conclusion,** in this pilot study, urinary and serum sCD163 levels were associated with kidney inflammatory activity in pediatric LN and differed from levels in extrarenal SLE and non-inflammatory CKD. These results indicate that sCD163 may serve as an exploratory biomarker reflecting inflammatory activity in LN. However, due to the heterogeneity of lupus immunopathogenesis, no single biomarker can replace kidney histopathology. However, given the small sample size, pre-defined study groups, and absence of inflammatory non-LN comparators, these results cannot be generalized to diagnostic use. Larger, longitudinal studies including clinically heterogeneous cohorts and inflammatory glomerulonephritis are needed to further evaluate the biological and clinical relevance of sCD163 in LN.

## Data Availability

The datasets used and/or analyzed during the current study are available from the corresponding author on reasonable request.
